# ASO Author Reflections: Type 2 Diabetes Mellitus and Insulin-Dependence as Determinants of Adverse In-Hospital Outcomes After Partial or Radical Nephrectomy

**DOI:** 10.1245/s10434-026-19381-y

**Published:** 2026-03-06

**Authors:** Maximilian Filzmayer, Michele Nicolazzini, Calogero Catanzaro, Federico Polverino, Michele Petix, Leonardo Quarta, Jordan A. Goyal, Alessandro Volpe, Riccardo Schiavina, Nicola Longo, Gennaro Musi, Alberto Briganti, Shahrokh F. Shariat, Mike Wenzel, Marina Kosiba, Clara Humke, Fred Saad, Felix K.-H. Chun, Pierre I. Karakiewicz

**Affiliations:** 1 Cancer Prognostics and Health Outcomes Unit, Division of Urology, University of Montreal Health Center, Montréal, QC Canada; 2 Department of Urology, Goethe University Frankfurt, University Hospital, Frankfurt am Main, Germany; 3 Division of Urology, Department of Translational Medicine, Maggiore della Carità Hospital, University of Eastern Piedmont, Novara, Italy; 4 Division of Urology, Department of Oncology, University of Turin, Orbassano, Italy; 5 Department of Urology, St. Orsola-Malpighi Hospital, University of Bologna, Bologna, Italy; 6 Department of Neurosciences, Science of Reproduction and Odontostomatology, University of Naples Federico II, Naples, Italy; 7 Department of Urology, IEO European Institute of Oncology, IRCCS, Milan, Italy; 8 Department of Oncology and Haemato-Oncology, Università degli Studi di Milano, Milan, Italy; 9 Division of Experimental Oncology/Unit of Urology, URI, Urological Research Institute, IRCCS San Raffaele Scientific Institute, Milan, Italy; 10 Vita-Salute San Raffaele University, Milan, Italy; 11 Department of Urology, Comprehensive Cancer Center, Medical University of Vienna, Vienna, Austria; 12 Department of Urology, Weill Cornell Medical College, New York, NY USA; 13 Department of Urology, University of Texas Southwestern Medical Center, Dallas, TX USA; 14 Hourani Center of Applied Scientific Research, Al-Ahliyya Amman University, Amman, Jordan

## Past

Perioperative risk stratification in kidney cancer surgery remains challenging, particularly in patients with type 2 diabetes mellitus (T2DM).^1^ Although diabetes is frequently treated as a binary comorbidity, insulin dependence reflects more advanced metabolic disease and has been associated with worse outcomes in non-urologic surgical fields.^2^ However, its impact on short-term, in-hospital outcomes after partial nephrectomy (PN) or radical nephrectomy (RN) was unknown. In addition, temporal trends in insulin-dependent (ID) versus non-insulin-dependent (NID) T2DM among nephrectomy patients had not been systematically evaluated.^3^ Our study addressed these gaps by analyzing national temporal trends and assessing the independent association between T2DM subtypes and adverse in-hospital outcomes after PN and RN.^4^

## Present

In a large contemporary cohort from the National Inpatient Sample, ID-T2DM was uncommon but increased markedly over time.^4^ After propensity score matching and multivariable adjustment, ID-T2DM was consistently associated with higher rates of adverse in-hospital outcomes, with the strongest effects observed in patients undergoing PN.^4^ Absolute and relative risk increases were substantially greater for those with ID-T2DM than for those with NID-T2DM, indicating that insulin-dependence provides clinically meaningful risk stratification beyond the presence of diabetes alone.^4^ The more pronounced effect in PN may reflect the higher technical complexity of nephron-sparing surgery. These findings complement epidemiologic data showing a rising burden of insulin-treated diabetes and align with perioperative literature identifying diabetes as a determinant of short-term surgical morbidity.

## Future

Future studies should prospectively validate these findings using datasets with greater clinical detail, including glycemic control, diabetes duration, renal function, and contemporary antidiabetic therapies. Interventional trials are needed to determine whether targeted perioperative optimization in patients with ID-T2DM can reduce postoperative morbidity.^5^ Beyond localized kidney cancer, the relevance of ID-T2DM should also be examined in cytoreductive nephrectomy for metastatic disease and in nephroureterectomy for upper tract urothelial carcinoma, where surgical complexity and patient vulnerability may further amplify metabolic risk.
